# The potential use of supercritical carbon dioxide in sugarcane juice processing

**DOI:** 10.1038/s41538-023-00242-x

**Published:** 2024-01-13

**Authors:** Fernanda Cristina Pimenta, Talita Cristiane Krice Moraes, Gustavo Cesar Dacanal, Alessandra Lopes de Oliveira, Rodrigo Rodrigues Petrus

**Affiliations:** Universidade de São Paulo Faculdade de Zootecnia e Engenharia de Alimentos, Pirassununga/SP São Paulo, Brasil

**Keywords:** Technology, Applied microbiology

## Abstract

Sugarcane juice is a nutritious and energetic drink. For its processing, the use of supercritical carbon dioxide (SC-CO_2_) technology as an intervention potentially capable of rendering a high quality product can be considered. This study evaluated the combined effect of SC-CO_2_ and mild temperatures, primarily aiming for the reduction of endogenous microorganisms and enzymes in sugarcane juice (pH~5.5). Pressures (P) ranging from 74 to 351 bar, temperatures (T) between 33 and 67 °C, and holding times (t) between 20 and 70 min were tested in a central composite rotational design. Seventeen trials were performed, comprising three replicates at the central points. Counts of aerobic mesophiles, molds and yeasts, lactic acid bacteria and coliforms at 45 °C, determination of polyphenol oxidase (PPO) and peroxidase (POD) activities, and measurement of color parameters in freshly extracted and processed juice’s samples were carried out. The pH of fresh and processed juice varied between 4.6 and 6.0, and between 4.6 and 6.3, respectively. The number of decimal reductions achieved in mesophiles, molds and yeasts, lactic acid bacteria and coliforms varied between 0.1 and 3.9, 2.1 and 4.1, 0.0 and 2.1, and 0.3 to 2.5, respectively. The percentages of PPO reduction ranged from 3.51% to 64.18%. Regarding the POD, reductions between 0.27% and 41.42% were obtained. Color variations between fresh and processed samples varied between 2.0 and 12.3. As for mesophiles, molds and yeasts reduction, and soluble solids variation, none of the variables or their interactions were significant. In terms of polyphenol oxidase (PPO) reduction, only t was significant; however, T, t, and the interaction between them significantly affected the peroxidase (POD) reduction. In regards to pH variation, P, and the interaction between T and t were significant. P, T, t, and the interaction between T and t played a significant effect on color. The combination of mild temperatures and SC-CO_2_ can be potentially used for cane juice preservation.

## Introduction

Sugarcane juice is a nutritious and energetic green brownish beverage with low acidity (pH 4.8-5.5) and high water activity (Aw~0.99). The juice’s composition depends on the variety, cultivar, maturation stage, soil, climatic and agricultural conditions. Cane juice has a limited shelf life due to its rapid microbiological and enzymic deterioration^1–3^, and when processed in industrial plants is subjected to heat treatment in order to inactivate enzymes, spoilage and potentially pathogenic microorganisms. Thermal processing, depending on its intensity, may however damage the sensory, functional and nutritional juice’s quality^4^. During extraction, the juice is exposed to oxygen, a reactant for enzymic browning, catalyzed by polyphenol oxidase (PPO) and peroxidase (POD), so the enzymic inactivation guarantees a higher quality product^1^. Additionally, it is necessary to eliminate spoilage microorganisms, such as *Leuconostoc mesenteroides*, molds and yeasts. *L. mesenteroids* produces lactic acid and changes the juice’s viscosity during storage^1^.

The industrialization of cane juice is rapidly growing in Brazil, and over the last few years more than 10 brands of processed cane juice have been launched. The preservation technologies frequently combine acidification, chemical preservatives, heat treatment and refrigeration, and vary among brands. Nevertheless, the sensory quality of the products available on the market is questionable. As an alternative to heat treatment, the technology that employs supercritical carbon dioxide (SC-CO_2_) is a promising intervention to inactivate pathogens and spoilage enzymes and microorganisms. This non-thermal technique consists of exposing food or beverages to high pressure (beyond 74 bar), and the main advantage is the preservation of the food sensory attributes^1^. In contrast, conventional heat treatments may trigger the onset of nutritional and sensory losses. A supercritical fluid is defined as any substance maintained above its critical temperature and pressure. The critical temperature is the highest temperature at which a gas can be converted into a liquid by increasing pressure. Critical pressure is the highest pressure at which a liquid can be converted into a gas by increasing the temperature of the liquid^5^. In SC-CO_2_ treatment, the food is exposed to pressurized CO_2_ for a certain period of time. The supercritical fluid diffuses through the food, showing a microbicidal effect, whose intensity depends on the pressure and holding time^6–8^. The temperature and pressure that characterize the critical state/point of carbon dioxide are 31.1 °C and 73.8 bar^4^.

Gómez-López et al.^9^ reported the specific energy required by the pressure change technology (PCT) application. The energy consumption per unit mass of treated product has been estimated and compared to that required by conventional indirect thermal technology. The estimated specific energy consumption was respectively 162.6 kJ/kg for indirect thermal and 26.3 kJ/kg for PCT. On the other hand, completely non-thermal processes such as PCT do not involve any energy costs for product and equipment cooling. Therefore, the energy consumption involved in this technology is only due to product and inert gas pumping and compression. Vignali et al.^10^ stated a comparison of typical specific working energy costs for thermal and non-thermal treatments in terms of KJ per kilograms of processed product. Non-thermal approaches seem to offer the most effective alternative in terms of nutrients and fresh-like characteristics preservation as well as working energy costs saving. Nevertheless, the SC-CO_2_ treatment has not been considered in that study because the literature is scarce and the data available for microbial inactivation are very low in comparison to the other technologies. According to Bocker and Silva^11^, CO_2_ stands out as the best cost-benefit among supercritical fluids since it has a low cost and is non-toxic. The critical conditions of CO_2_ (73,8 Bar and 31 °C) are moderate compared to those of other fluids used in the supercritical state. These moderate conditions reduce the process energy expenditure and promote less damage to the nutritional properties of the food matrices.

Many studies address the direct injection application of SC-CO_2_ in the inactivation of enzymes and microorganisms in fruit juices^1,12–14^. The SC-CO_2_ proved to be efficient in the inactivation of microorganisms and enzymes. Additionally, the low toxicity and cost are important advantages of CO_2_ since it is naturally found in the atmosphere. The use of SC-CO_2_ under mild conditions is a technique that, when used in juices, allows greater preservation of thermally unstable constituents, such as phenolic compounds, flavonoids and anthocyanins^15^. Nevertheless, no work targeting the stabilization of SC-CO_2-_treated cane juice has been found. This study was primarily conducted to evaluate the combined effect of mild temperatures and SC-CO_2_ on microorganisms and enzymes in cane juice.

## Results and discussion

### Physicochemical tests

Table 1 exhibits the pH and soluble solids values determined in raw and processed cane juice.

**Table 1:** Physicochemical parameters of raw and SC-CO_2_-treated cane juice.

Trial	Treatment	pH	Soluble solids (°Brix)
1	raw	5.4 ± 0.1	25.3 ± 0.0
	213 bar/50 °C/45 min	5.3 ± 0.0	24.9 ± 0.0
	∆	−0.1	−0.4
2	raw	5.2 ± 0.1	24.2 ± 0.1
	130 bar/40 °C/30 min	5.0 ± 0.1	24.0 ± 0.1
	∆	−0.2	−0.2
3	raw	4.6 ± 0.0	22.1 ± 0.1
	295 bar/40 °C/30 min	4.6 ± 0.1	21.8 ± 0.1
	∆	0.0	−0.3
4	raw	5.4 ± 0.1	25.2 ± 0.1
	130 bar/60 °C/30 min	5.1 ± 0.1	25.0 ± 0.1
	∆	−0.3	−0.2
5	raw	5.4 ± 0.1	24.3 ± 0.0
	295 bar/60 °C/30 min	5.2 ± 0.1	24.0 ± 0.1
	∆	−0.2	−0.3
6	raw	5.4 ± 0.0	23.5 ± 0.1
	213 bar/50 °C/45 min	5.4 ± 0.0	23.2 ± 0.1
	∆	0.0	−0.3
7	raw	5.4 ± 0.1	23.6 ± 0.1
	130 bar/40 °C/60 min	5.0 ± 0.1	23.2 ± 0.1
	∆	−0.4	−0.4
8	raw	5.3 ± 0.1	23.7 ±
	295 bar/40 °C/60 min	5.1 ±	23.6 ±
	∆	−0.2	-0.1
9	raw	4.6 ± 0.1	21.4 ± 0.1
	130 bar/60 °C/60 min	4.4 ± 0.0	21.2 ± 0.1
	∆	−0.2	−0.2
10	raw	5.3 ± 0.1	18.7 ± 0.1
	295 bar/60 °C/60 min	5.5 ± 0.0	18.7 ± 0.1
	∆	0.2	0.0
11	raw	5.5 ± 0.1	20.6 ± 0.1
	213 bar/50 °C/45 min	5.6 ± 0.1	20.4 ± 0.0
	∆	0.1	−0.2
12	raw	6.0 ± 0.1	19.0 ± 0.1
	74 bar/ 50 °C/ 45 min	6.3 ± 0.0	18.7 ± 0.1
	∆	0.3	−0.3
13	raw	5.6 ± 0.1	20.9 ± 0.1
	351 bar/50 °C/45 min	5.4 ± 0.0	20.8 ± 0.1
	∆	−0.2	−0.1
14	raw	5.4 ± 0.1	20.4 ± 0.1
	213 bar/33 °C/45 min	5.2 ± 0.0	20.1 ± 0.1
	∆	−0.2	−0.3
15	raw	5.6 ± 0.1	19.9 ± 0.1
	213 bar/67 °C/45 min	5.5 ± 0.1	19.9 ± 0.0
	∆	−0.1	0.0
16	raw	5.6 ± 0.1	18.6 ± 0.1
	213 bar/50 °C/20 min	5.6 ± 0.0	18.6 ± 0.1
	∆	0.0	0.0
17	raw	5.3 ± 0.1	18.5 ± 0.1
	213 bar/50 °C/70 min	5.4 ± 0.0	18.2 ± 0.1
	∆	0.1	−0.3

Mean values of 3 replicates ± standard deviation.

Δ - variation.

The pH values ranged from 4.6 to 6.0 in the raw juice and between 4.4 and 6.3 for the processed one. The treatments reduced up to 0.4 units in the pH; however, the pH from trials 3 (295 bar/40 °C/30 min), 6 (213 bar/50 °C/45 min) and 16 (213 bar/50 °C/20 min) remained unchanged. The variety of cane, type of soil, fertilization, climatic conditions, degree of maturity, harvesting and extraction methods are important factors to be considered in the variation of juice’s pH. Bomdespacho et al.^16^ evaluated different cultivars of raw cane juice and reported an average pH equivalent to 5.05. This data is close to the values found in most treatments performed in the present study, with the exception of trials 3, 9 and 12.

Regarding the soluble solids content, variations between 18.5 and 25.3 °Brix were determined in the raw juice, and between 18.2 and 25.0 for the processed beverage. The variations (Δ) in this parameter caused by the treatment ranged between 0.0 and 0.4. With the exception of trials 15 and 16, there was a reduction in this parameter. These phenomena may be related to the variation of the treatments submitted, as well as to the batch used on the day of the respective trials. Bomdespacho et al.^16^ reported an average of 21.2 °Brix in fresh juice extracted from different cultivars. This result is in the range obtained in this study. In all 17 trials, no meaningful variations (Δ ≤ 0.4) were observed between processed and raw juice. These findings are positive as they lead to the hypothesis that there was no significant difference between the pH values and soluble solids after the treatments applied.

### Microbiological assays

Table 2 reports the microbial counts in raw and SC-CO_2_-treated cane juice as well as the log reduction achieved in each trial.

**Table 2:** Microbial counts (logCFU/mL) in raw and SC-CO_2_-treated cane juice.

Trial	Treatment	Coliforms (45 °C)	Mesophiles	Lactic bacteria	Molds and yeasts
	raw	1.5 ± 0.2	3.6 ± 0.1	1.0 ± 0.0	3.5 ± 0.3
1	213 bar/50 °C/45 min	<1_est_	1.8 ± 0.1	<1_est_	1.1 ± 0.1
	**log red**	>0.5 _est_	1.8	-	2.4
	raw	<1_est_	3.1 ± 0.4	2.6 ± 0.3	3.5 ± 0.3
2	130 bar/40 °C/30 min	<1_est_	2.7 ± 0.1	1.2 ± 0.2	1.4 ± 0.2
	**log red**	-	0.4	1.4	2.1
	raw	<1_est_	4.6 ± 0.3	1.3 ± 0.1	2.4 ± 0.2
3	295 bar/40 °C/30 min	<1_est_	2.6 ± 0.3	< 1_est_	0.3 ± 01
	**log red**	-	2.0	> 0.3 _est_	2.1
	raw	2.5 ± 0.1	4.6 ± 0.2	3.0 ± 0.2	4.2 ± 0.4
4	130 bar/60 °C/30 min	0.7 ± 0.2	1.6 ± 0.1	1.0 ± 0.2	1.0 ± 0.1
	**log red**	1.8	3.0	2.0	3.2
	raw	1.3 ± 0.3	3.7 ± 0.1	3.1 ± 0.2	3.8 ± 0.5
5	295 bar/60 °C/30 min	<1_est_	1.5 ± 0.1	1.5 ± 0.2	1.6 ± 0.3
	**log red**	>0.3	2.2	1.6	2.2
	raw	<1_est_	4.9 ± 0.3	3.0 ± 0.3	4.1 ± 0.3
6	213 bar/50 °C/45 min	<1_est_	1.7 ± 0.2	1.6 ± 0.3	0.9 ± 0.2
	**log red**	-	3.2	1.4	3.2
	raw	2.3 ± 0.1	2.7 ± 0.2	2.2 ± 0.2	4.9 ± 0.3
7	130 bar/40 °C/60 min	<1_est_	<1_est_	2.1 ± 0.2	1.7 ± 0.2
	**log red**	>1.3_est_	>1.7_est_	0.1	3.2
	raw	<1_est_	6.0 ± 0.3	3.5 ± 0.2	4.1 ± 0.3
8	295 bar/40 °C/60 min	<1_est_	2.1 ± 0.2	1.4 ± 0.2	1.8 ± 0.2
	**log red**	-	3.9	2.1	2.3
	raw	3.5 ± 0.3	4.7 ± 0.3	< 1_est_	5.4 ± 0.2
9	130 bar/60 °C/60 min	2.2 ± 0.1	1.7 ± 0.1	< 1_est_	2.6 ± 0.1
	**log red**	1.3	3.0	-	2.8
	raw	5.2 ± 0.1	3.0 ± 0.1	4.0 ± 0.1	4.9 ± 0.6
10	295 bar/60 °C/60 min	2.9 ± 0.3	2.0 ± 0.3	2.2 ± 0.1	1.5 ± 0.1
	**log red**	2.3	1.0	1.8	3.4
	raw	3.9 ± 0.1	2.0 ± 0.2	2.1 ± 0.2	4.9 ± 0.4
11	213 bar/50 °C/45 min	1.4 ± 0.3	1.9 ± 0.1	0.7 ± 0.1	2.7 ± 0.2
	**log red**	2.5	0.1	1.4	2.2
	raw	3.3 ± 0.1	5.5 ± 0.6	0.7 ± 0.2	4.8 ± 0.4
12	74 bar/ 50 °C/ 45 min	2.3 ± 0.4	3.2 ± 0.2	0.4 ± 0.2	1.9 ± 0.1
	**log red**	1.0	2.3	0.3	2.9
	raw	3.4 ± 0.2	5.6 ± 0.1	3.9 ± 0.2	4.2 ± 0.4
13	351 bar/50 °C/45 min	2.5 ± 0.2	3.6 ± 0.3	2.8 ± 0.2	1.8 ± 0.3
	**log red**	0.9	2.0	1.1	2.4
	raw	3.3 ± 0.1	5.6 ± 0.3	1.2 ± 0.1	5.1 ± 0.5
14	213 bar/33 °C/45 min	2.8 ± 0.2	2.8 ± 0.1	< 1_est_	2.6 ± 0.3
	**log red**	0.5	2.8	> 0.2_est_	2.5
	raw	1.8 ± 0.2	5.9 ± 0.5	2.5 ± 0.4	4.7 ± 0.1
15	213 bar/67 °C/45 min	<1_est_	4.1	< 1_est_	0.6 ±
	**log red**	>0.8_est_	1.8	0.0	4.1
	raw	2.8 ± 0.3	5.3 ± 0.5	3.7 ± 0.1	5.3 ± 0.2
16	213 bar/50 °C/20 min	0.8 ± 0.1	3.2 ± 0.2	3.5 ± 0.3	1.8 ± 0.2
	**log red**	2.0	2.1	0.2	3.5
	raw	2.9 ± 0.2	4.8 ± 0.1	3.6 ± 0.3	4.3 ± 0.2
17	213 bar/50 °C/70 min	0.8 ± 0.1	2.8 ± 0.2	2.8 ± 0.1	1.6 ± 0.3
	**log red**	2.1	2.0	0.8	2.7

Mean values of 3 replicates ± standard deviation.

est – estimate count (under detection limit).

The results exhibited in Table 2 show the potential of SC-CO_2_ in the reduction of contaminants in raw cane juice. The reductions achieved by the different trinomials were 2.5 log for coliforms, 3.9 log for aerobic mesophiles, 2.1 log for lactic acid bacteria, and 4.1 log for molds and yeasts.

The lactic bacteria counts in raw juice varied between 1.0 and 4.0 logCFU/mL. For the processed sample, counts ranged from <1.0_est_ to 3.5 logCFU/mL; comparison with data from other studies was not possible, once counts were carried out after cane fermentation, as reported by Silva et al.^17^, who performed counts after 3, 11 and 24 h of fermentation. The lactic bacteria contamination, such as *Leuconostoc mesenteroides* and some species of the *Lactobacillus*, can trigger the synthesis of dextrans (polysaccharides formed by glucose units) (Koblitz)^18^, forming gums in the juice, leading to its rejection. The discrepancy among counts within the same group of microorganisms in raw juice may be attributed to failures in the hygiene procedures of the raw material, utensils and/or equipment used in the extraction. This event is usual when it comes to street vending.

According to Prati, Moretti and Cardello^19^, mesophilic counts above 6.0 logCFU/mL may be related to hygienic-sanitary deficiencies in the extraction and/or storage of cane juice. In this study both the raw and processed juice exhibited counts within the range 1.6–6.0 logCFU/mL. For molds and yeasts, counts were between 2.4 and 5.4 logCFU/mL; Jay^20^ holds that values above 3.0 logCFU/mL can cause undesirable changes.

The efficiency of SC-CO_2_ treatment on microbial inactivation is associated with the modification of intracellular and extracellular pH, and also the length of time CO_2_ diffuses into the cells. Therefore, the holding time of treatment greatly impacts the microbial inactivation rate^4^.

Dhansu et al.^21^ pasteurized cane juice at 65 °C/25 min, and stored it under refrigeration, achieving a shelf life of 60 days. Oliveira et al.^1^ pasteurized the juice at 70 °C/25 min; the lactic acid bacteria counts in raw and processed cane juice were (5.9 and 1.3) logCFU/mL respectively, reaching 4.6 log reduction. The molds and yeasts’ counts in raw and processed juice were (6.1 and 1.7) logCFU/mL respectively. Gomes et al.^22^ optimized the time x temperature binomial used in the pasteurization of whole cane juice; temperatures and holding times ranging between 78 and 92 °C, and from 16 to 44 s, were tested. Regarding the reduction of microorganisms, the treatment at 90 °C/40 s was the most efficient, achieving 4.6 log reductions for mesophiles. For molds and yeasts, 3.2 log reductions were reached.

Hart et al.^23^ reported the application of SC-CO_2_ in the inactivation of spores in foods, highlighting how this technique can be more efficient in preserving nutritional and sensory characteristics as compared to high hydrostatic pressure techniques and thermal methods at high temperatures. The action of SC-CO_2_ occurs through disruption of the cell wall, coating, cortex and membranes, and degradation of proteins. More in-depth studies on larger scales are needed to disseminate this technology in the processing of fruits and juices.

Eggleston^24^ reported the microbiological, enzymic and chemical deterioration (acid degradation) of sucrose in cane juice. The findings indicated that the growth of microorganisms is relevant for sucrose degradation. After 14 h, the largest contribution was microbiological, accounting for 93% of losses, while enzymatic degradation contributed with 5.7% of losses and chemical degradation with 1.3%.

### Enzymic tests

The endogenous enzymes activity (polyphenol oxidase and peroxidase) as well as the percentages of reduction achieved by different treatments are shown in Table 3.

**Table 3:** Polyphenol oxidase (PPO) e peroxidase (POD) activities (U) in raw and SC-CO_2_-treated cane juice.

Trial	Treatment	PPO	POD
	raw	11.4 ± 0.0	226.7 ± 0.1
1	213 bar/50 °C/45 min	7.7 ± 0.2	198.0 ± 0.5
	**red (%)**	32.5	12.7
	raw	11.0 ± 0.5	245.1 ± 0.5
2	130 bar/40 °C/30 min	5.4 ± 0.5	202.2 ± 2
	**red (%)**	50.9	17.5
	raw	11.0 ± 0.1	238.4 ± 1
3	295 bar/40 °C/30 min	6.6 ± 0.1	202.2 ± 2
	**red (%)**	40.0	15.2
	raw	11.0 ± 0.1	231.6 ± 7
4	130 bar/60 °C/30 min	6.1 ± 0.3	202.2 ± 2
	**red (%)**	44.5	12.7
	raw	11.0 ± 0.1	202.2 ± 2
5	295 bar/60 °C/30 min	3.9 ± 0.0	192.9 ± 0.3
	**red (%)**	64.5	4.6
	raw	11.4 ± 0.0	235.6 ± 2
6	213 bar/50 °C/45 min	7.7 ± 0.3	190.7 ± 0.0
	**red (%)**	32.5	19.1
	raw	11.4 ± 0.0	232.2 ± 6
7	130 bar/40 °C/60 min	9.4 ± 0.3	183.8 ± 4.1
	**red (%)**	17.5	20.8
	raw	11.4 ± 0.0	231.1 ± 8
8	295 bar/40 °C/60 min	8.0 ± 0.2	215.1 ± 3
	**red (%)**	29.8	6.9
	raw	10.0 ± 0.2	56.1 ± 4
9	130 bar/60 °C/60 min	9.3 ± 2.0	34.0 ± 7
	**red (%)**	7.0	39.4
	raw	3.4 ± 0.6	56.1 ± 4
10	295 bar/60 °C/60 min	2.5 ± 0.4	34.0 ± 7
	**red (%)**	26.5	39.4
	raw	2.3 ± 0.6	8.4 ± 2
11	213 bar/50 °C/45 min	0.8 ± 0.4	7.2 ± 2
	**red (%)**	63.2	14.3
	raw	1.8 ± 0.6	8.1 ± 2
12	74 bar/ 50 °C/ 45 min	0.7 ± 0.4	6.7 ± 2
	**red (%)**	61.1	17.3
	raw	2.4 ± 0.5	8.1 ± 0.2
13	351 bar/50 °C/45 min	0.9 ± 0.5	5.6 ± 2
	**red (%)**	62.5	30.9
	raw	18.0 ± 0.7	7.3 ± 2
14	213 bar/33 °C/45 min	17.4 ± 0.5	7.2 ± 2
	**red (%)**	3.3	1.4
	raw	21.9 ± 0.7	8.8 ± 2
15	213 bar/67 °C/45 min	20.5 ± 0.3	5.2 ± 0.1
	**red (%)**	6.4	40.9
	raw	2.7 ± 0.2	7.1 ± 2
16	213 bar/50 °C/20 min	1.2 ± 0.0	7.1 ± 2
	**red (%)**	55.6	0.0
	raw	3.4 ± 0.6	5.1 ± 0.1
17	213 bar/50 °C/70 min	2.1 ± 0.4	5.0 ± 0.4
	**red (%)**	38.2	2.0

Mean values of 3 replicates ± standard deviation.

Red reduction.

The results exhibited in Table 3 suggest the potential of SC-CO_2_ combined with mild temperatures to inactivate the endogenous enzymes that are responsible for the degradation of the color, flavor and the nutritional values of cane juice. The percentages of PPO (3.3–64.5%) and POD (0.0–40.9%) reduction varied widely. The trinomial applied in trial 5 (295 bar/60 °C/30 min) reached the greatest PPO inactivation (64.5%), suggesting that temperature had a more significant effect on the percentage of reduction; however, this hypothesis will be confirmed in the light of the statistical analysis of the effects of the variables studied. POD exhibited greater resistance to the treatments in most trials. The trinomial applied in trial 15 (213 bar/67 °C/45 min) reached the highest percentage of inactivation (40.9%). Marszałek et al.^12^ studied the effect of supercritical carbon dioxide on PPO and POD in mushroom and radish; surprisingly, PPO was more resistant to temperature and pressure than POD. In this study, similar result was observed in trials 7, 9, 10 and 15, i.e., PPO was more resistant to SC-CO_2_. In most trials, however, the percentage of POD reduction in the juice was lower than PPO. This finding suggests that the type of food matrix also influences the impact of the technology that is applied. The food matrix can interfere with the intermolecular bonds of the two enzymes depending on the amount of water in the medium, since the impact of pressure on intra and intermolecular interactions can also be correlated with the ability of the functional groups of the enzymes to interact with water (Marszałek et al.)^25^. The deactivation of enzymes exposed to high temperatures and prolonged times is explained by changes in the tertiary and secondary structures of the protein. The thermal stability of enzymes depends on a number of factors such as source, species, nature of the food matrix (Iqbal et al.)^26^.

### Color parameters

The color parameters instrumentally measured in cane juice are presented in Table 4.

**Table 4:** Color parameters determined in raw and SC-CO_2_-treated cane juice.

Trial	Treatment	L*	a*	b*	chroma	°hue
1	raw	33.67 ± 0.02	8.71 ± 0.01	29.95 ± 0.02	31.2	73.8
	213 bar/50 °C/45 min	37.91 ± 0.01	6.25 ± 0.02	27.92 ± 0.02	28.6	77.4
2	raw	34.17 ± 0.02	10.5 ± 0.01	32.55 ± 0.01	34.2	72.1
	130 bar/40 °C/30 min	35.75 ± 0.03	9.37 ± 0.02	30.02 ± 0.01	31.4	72.7
3	raw	32.26 ± 0.02	10.5 ± 0.02	26.03 ± 0.01	28.1	68.1
	295 bar/40 °C/30 min	34.92 ± 0.01	9.02 ± 0.01	26.40 ± 0.01	27.9	71.1
4	raw	36.75 ± 0.01	6.19 ± 0.02	36.71 ± 0.03	37.2	80.4
	130 bar/60 °C/30 min	37.77 ± 0.02	6.27 ± 0.02	34.55 ± 0.02	35.1	79.7
5	raw	38.93 ± 0.03	4.61 ± 0.01	35.44 ± 0.05	35.7	82.6
	295 bar/60 °C/30 min	44.14 ± 0.02	3.96 ± 0.05	37.62 ± 0.03	37.6	84.0
6	raw	69.03 ± 0.10	0.96 ± 0.0	36.60 ± 0.02	36.6	88.5
	213 bar/50 °C/45 min	63.14 ± 0.02	4.62 ± 0.01	43.95 ± 0.01	44.2	84.0
7	raw	40.67 ± 0.01	4.89 ± 0.01	38.58 ± 0.02	38.9	82.8
	130 bar/ 40 °C/ 60 min	42.55 ± 0.02	4.89 ± 0.01	37.84 ± 0.01	38.2	82.6
8	raw	72.92 ± 0.01	1.22 ± 0.02	36.71 ± 0.02	36.7	88.1
	295 bar/40 °C/60 min	73.06 ± 0.01	4.04 ± 0.01	42.28 ± 0.02	42.5	84.5
9	raw	71.09 ± 0.02	2.64 ± 0.04	35.90 ± 0.03	36.0	85.8
	130 bar/60 °C/60 min	60.91 ± 0.02	3.97 ± 0.02	39.33 ± 0.02	39.5	84.2
10	raw	37.02 ± 0.01	16.09 ± 0.0	45.84 ± 0.01	40.6	70.7
	295 bar/60 °C/60 min	50.14 ± 0.02	14.74 ± 0.0	50.67 ± 0.02	52.8	73.8
11	raw	31.48 ± 0.02	4.60 ± 0.03	30.29 ± 0.03	30.6	81.4
	213 bar/50 °C/45 min	32.53 ± 0.01	5.80 ± 0.03	24.75 ± 0.02	25.4	76.8
12	raw	55.16 ± 0.02	5.14 ± 0.02	41.59 ± 0.02	41.9	83.0
	74 bar/ 50 °C/ 45 min	49.73 ± 0.02	6.78 ± 0.02	34.34 ± 0.01	35.0	78.8
13	raw	49.15 ± 0.01	5.65 ± 0.01	44.44 ± 0.01	44.8	82.8
	351 bar/50 °C/45 min	51.37 ± 0.01	4.33 ± 0.02	37.39 ± 0.05	37.6	83.4
14	raw	60.81 ± 0.02	4.94 ± 0.01	44.73 ± 0.01	44.9	83.7
	213 bar/33 °C/45 min	66.36 ± 0.02	4.05 ± 0.04	38.75 ± 0.02	39.0	84.0
15	raw	58.49 ± 0.01	3.05 ± 0.05	40.05 ± 0.02	40.2	85.6
	213 bar/67 °C/45 min	52.32 ± 0.02	4.25 ± 0.03	36.51 ± 0.01	36.8	83.4
16	raw	52.49 ± 0.03	6.53 ± 0.05	45.88 ± 0.02	46.3	81.9
	213 bar/50 °C/20 min	52.82 ± 0.02	6.63 ± 0.04	41.09 ± 0.02	41.6	80.8
17	raw	63.63 ± 0.01	4.84 ± 0.03	46.17 ± 0.05	46.4	84.0
	213 bar/50 °C/70 min	65.70 ± 0.04	4.58 ± 0.02	42.14 ± 0.02	42.4	83.8

L* (lightness) = 0 (black). 100 (white). +a* = red. −a* = green. +b* = yellow. −b* = blue.

Mean values of 3 replicates ± standard deviation.

$${\rm{Chroma}}={({{\rm{a}}}^{* 2}+{{\rm{b}}}^{* 2})}^{\frac{1}{2}}\, ^\circ {\rm{hue}}=\arctan \left(\frac{{{\rm{b}}}^{* }}{{{\rm{a}}}^{* }}\right)$$.

The parameter L*, which represents lightness, varied widely for raw (32.3–72.9) and processed (32.5–73.1) juice. Most treatments positively influenced the lightness of the juice samples, which is most likely related to enzymic inactivation (Table 3). This result could favor the juice’s sensory acceptance, assuming the consumers prefer a lighter drink. Similarly, there was a great variation in the a* parameter for fresh (1–16.1) and processed (4.0–14.7) juice. The b* parameter also varied considerably for raw (26.0–46.2) and processed (24.8–50.7) samples. Regarding the chroma parameter (C*), significant variations were also observed for raw (28.1–46.4) and processed (25.4–52.8) juice. Chroma correlates to saturation, characterizing the sample’s color as “vivid” or opaque (dull). This attribute is independent of lightness and °hue. Saturation ranges from purple-red to green, and increases from the center (0) to the edge of the color wheel. Oliveira et al.^1^ stated that low C* values represent gray, and values close to 60 represent vivid colors; they found C* values close to 9 (more neutral color) for raw cane juice, in contrast to the present study. Meerod^27^ studied different cultivars of raw material, which showed divergent colors at various levels. Following the same behavior as the previous parameters, the hue angle also showed great variation for raw (68.1–88.5) and processed (71.1–84.5°) juices. Hue, measured in degrees, classifies color (green, yellow, blue, etc.). The ranges determined in this study are positioned in the first quadrant of the color circle, and can be classified between yellow-red and yellow. The wide variation ranges in the color parameters can be explained by the variability inherent to the raw material; juice samples extracted on different days, from different stalks, were used during the course of this research.

**Fig. 1:**
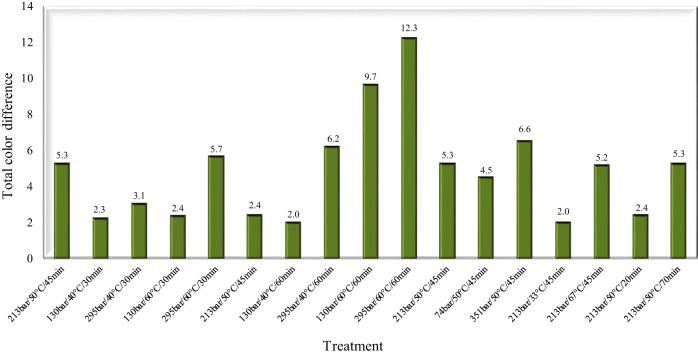
Total color difference (TCD) between raw and SC-CO_2_-processed cane juice.

Figure 1 illustrates the total color difference (TCD) between raw and processed cane juice.

The highest (12.3) and lowest (2.0) values of total color difference (∆E*) were determined for juice treated at 295 bar/ 60 °C/ 60 min and (130 bar/ 40 °C/ 60 min and 213 bar/ 33 °C/45 min), respectively. Bernard et al.^28^ states that ∆E* values less than 3 cannot be easily detected by the human eye, and values greater than 12 represent different color “spaces”. Therefore, of the 17 tests carried out, only six preserved the original color of the juice, in terms of its sensory perception.

### Statistical analysis

**Fig. 2:**
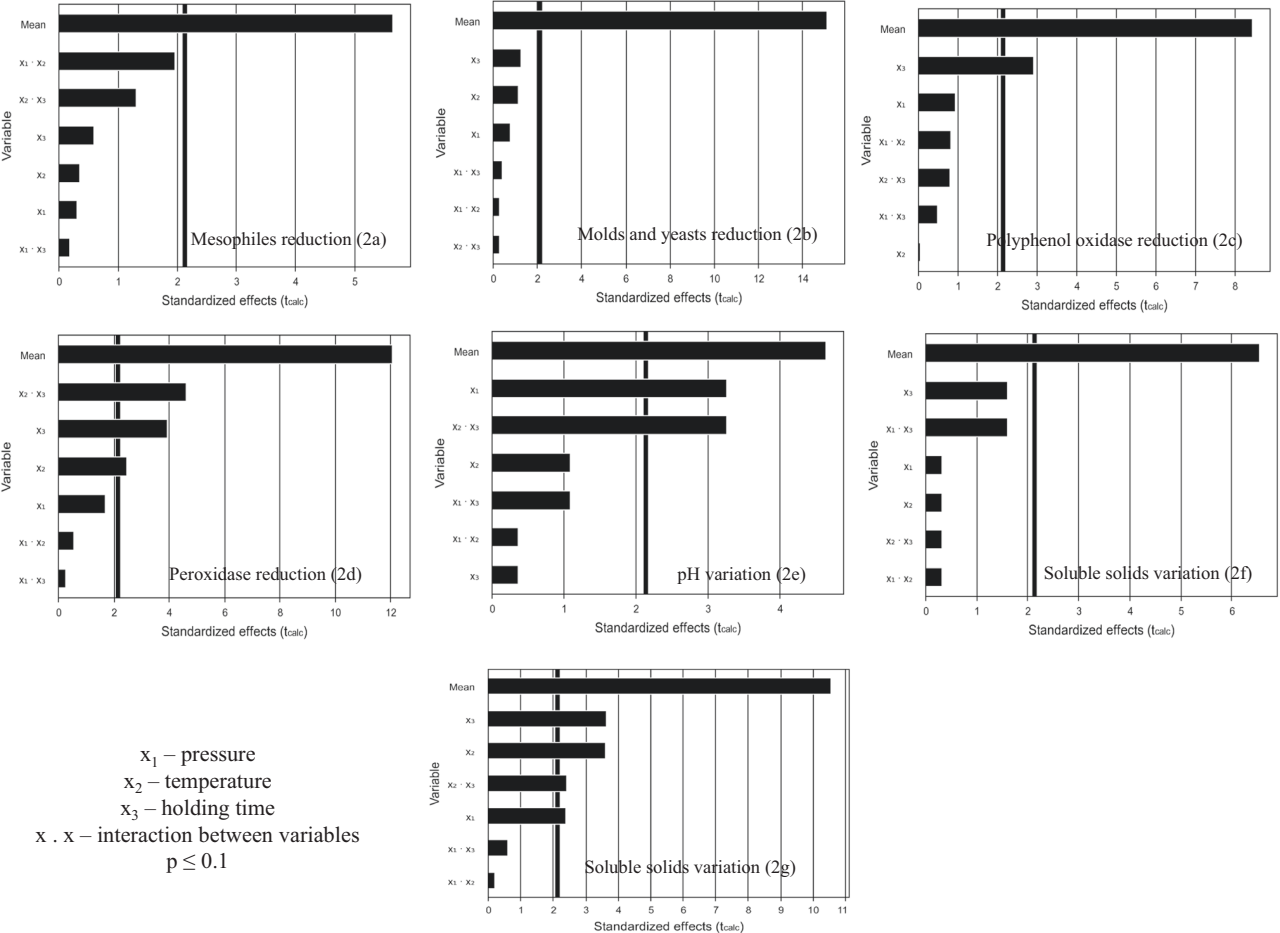
Pareto diagrams for cane juice treated with SC-CO_2_. Pareto diagrams for mesophiles reduction (2a), molds and yeasts reduction (2b), polyphenol oxidase reduction (2c), peroxidase reduction (2d), pH variation (2e), soluble solids variation (2 f) and total color difference (2 g).

Because the log reduction in coliforms and lactic bacteria could not be calculated in some trials (Table 3), these responses were not subjected to the statistical analysis. Figure 2 demonstrates the Pareto diagrams, built to investigate which parameters/variables (pressure/P/x_1_, temperature/T/x_2_, holding time/t/x_3_) were significant (*p* ≤ 0.1) in the studied responses. The terms that were not statistically significant were incorporated into the lack-of-fit to calculate the coefficient of determination (R^2^).

As for mesophiles, molds and yeasts reduction, and soluble solids variation, none of variables or their interactions were significant. In terms of polyphenol oxidase (PPO) reduction, only t (x_3_) was significant; however, the parameters T (x_2_), t (x_3_), and the interaction between them (x_2_.x_3_) played a significant effect on the peroxidase (POD) reduction. In regards to pH variation, P (x_1_) and the interaction between T and t (x_2_.x_3_) were significant. Finally, P, T, t, and the interaction between T and t were significant in the total color difference.

Only the significant variables were encompassed into the mathematical model, whose statistical significance was evaluated through analysis of variance (ANOVA). Table 5 exhibits the ANOVA carried out for the models (1st and 2nd orders) generated for the responses *POD reduction* and *total color difference* (TDC); the models for other responses were not statistically significant (*p* > 0.1). The coded predicted models obtained for the aforementioned responses are represented by Eqs. 1 and 2.

1 $${{\rm{Y}}}_{1}=18.56+4.46\,{x}_{2}+7.09\,{x}_{3}+8.30\,{x}_{2}\,{x}_{3}$$

Y_1_ – POD reduction (%)

x_2_ – Temperature (T)

x_3_ – holding time (t)

2 $${{\rm{Y}}}_{2}=5.15+1.36\,{x}_{1}+2.06\,{x}_{2}+2.09\,{x}_{3}+1.39\,{x}_{2}\,{x}_{3}$$

Y_2_ – Total color difference

x_1_ – Pressure (P)

x_2_ – Temperature (T)

x_3_ – holding time (t)

**Table 5:** Analysis of variance (*p* ≤ 0.1) for peroxidase reduction and total color difference in cane juice.

Response	Variation source	Sum of squares	Degrees of freedom	Mean square	*F* value	
					F_calc_	F_tab_
Peroxidase reduction 1^st^ order	Regression	1112.0	3	370.7	13.8	3.1
	Residual	188.4	7	26.9		
	Total	1300.4				
	R^2^	0.86				
Peroxidase reduction 2^nd^ order	Regression	1356.7	2	678.3	6.2	2.73
	Residual	1536.4	14	109.7		
	Total	2893.1				
	R^2^	0.47				
Total color difference 1^st^ order	Regression	99.1	4	24.8	13.1	3.2
	Residual	11.5	6	1.9		
	Total	110.6				
	R^2^	0.90				
Total color difference 2^nd^ order	Regression	99.8	4	25.0	10.0	2.5
	Residual	29.5	12	2.5		
	Total	129.3				
	R^2^	0.77				

For practical purposes, it is desirable that the fitted model be as simple as possible and contain the smallest possible number of parameters without giving up the quality assured in the careful selection of the experimental design. The models herein presented were re-parameterized/reduced because the parameters with little or no influence on the outcome of the final fit were excluded.

Regarding the response *POD reduction*, Table 5 shows that the 1^st^ order model (R^2^ = 0.86) better fitted to experimental data than the 2^nd^ order model (R^2^ = 0.47). For both orders, F_calc_ was greater than F_tab_. Similarly, as for the *total color difference* (TCD), the 1st order mathematical model (R^2^ = 0.90) best fitted to experimental data. The coded Eqs. 1 and 2 can be used to predict the percentage of POD reduction and the TCD that can be achieved in cane juice processed under the same conditions of this study. The coded model is that whose regression coefficients are obtained from the matrix of coded variables (−α, −1, 0, +1, +α). Given this, to obtain a predicted value from the model one must replace the values in the coded equation. In contrast, if using real values for the variables in the model, the predicted value may be incorrect and even absurd. Of particular relevance is the claim that the first order mathematical models hereby presented (Eqs. 1 and 2) are only valid in a range of pressure from 130 to 295 bar, temperature from 40 to 60 min, and holding time between 30 and 60 min (Table 6).

**Table 6:** Actual and coded levels tested in the central composite rotational design (CCRD) for cane juice processing with SC-CO_2_.

Variable	code	−1.68 (-α)	−1	0	+1	+1.68 ( + α)
Pressure (bar)	x_1_	74	130	213	295	351
Temperature (°C)	x_2_	33	40	50	60	67
Time (min)	x_3_	20	30	45	60	70

(−1.68) lower axial point, (−1) lower level, (0) central point, (+1) upper level, (+1.68) upper axial point. α = (2^n^)^1/4^ = 1.68. *n* = number of variables (3).

**Fig. 3:**
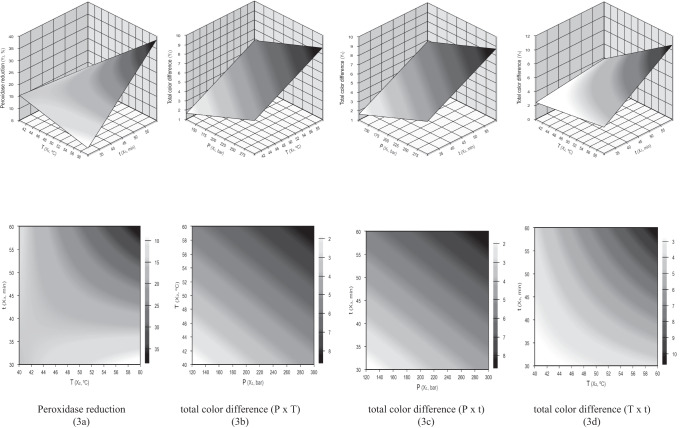
Response surfaces and contour curves for peroxidase reduction (3a), and total difference color (3b, 3c, 3d) in cane juice treated with SC-CO_2_. Response surfaces.

Figure 3 depicts the response surfaces and contour curves that represent Eqs. 1 and 2. By analyzing the surface for POD reduction, one can identify the existence of an optimal range for the temperature (57–60 °C) and holding time (56–60 min), regardless the pressure (in the range 130–295 bar). As for TDC, the ranges 130–150 bar, 40–43 °C and 30–35 min, within which the color difference between raw and processed juice is minimal, represent the optimal conditions in this experiment. This is of much greater interest than a simple point value, because it provides information about the “robustness” of the process, and most notably, it is the variation in pressure, temperature and holding time that may be permitted around optimal values which still maintains the process under optimized conditions. This finding is fundamental for the control engineer to define and maintain the pressure, temperature and time sensors and controller levels. This directly affects viability and process implementation (Rodrigues and Iemma)^29^.

A fact worth highlighting is that studies addressing the use of SC-CO_2_ in cane juice processing have not been found. In this way, data comparison could not be made. The combination of supercritical carbon dioxide and mild temperatures exhibited a meaningful effect on microorganism’s reduction in sugarcane juice, under the conditions of this study. Endogenous enzymes that deteriorate the juice’s quality were partially inactivated. None of variables (pressure/P, temperature/T, holding time/t) or their interactions were significant in mesophiles, molds and yeasts reduction, or soluble solids variation. In terms of polyphenol oxidase (PPO) reduction, only t was significant; however, T, t and the interaction between them played a significant effect on the peroxidase (POD) reduction. In regards to pH variation, P and the interaction between T and t were significant. Finally, P, T, t, and the interaction between T and t were significant in the total color difference. The optimal parameters (P, T and t) determined in this study varied for different responses. The combination of mild temperatures and SC-CO_2_ can be potentially used for cane juice preservation.

## Methods

**Fig. 4:**
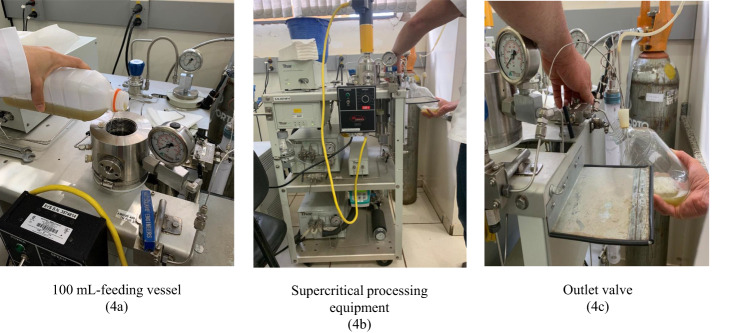
Cane juice processing (4a. 100 mL-feeding vessel, 4b. supercritical processing equipment, 4c. outlet valve). Juice processing.

The cane juice was procured from a local vendor, in the city of Pirassununga/SP-Brazil. The freshly extracted juice was collected in a plastic bottle by the vendor, kept on ice in an isothermal container, and rapidly transported to the laboratory in the Food Engineering Department at the University of Sao Paulo. The juice’s sample was divided into two parts and collected in previously sterilized glass bottles with screw caps. One fraction was used as a control (unprocessed juice) and the other one was treated with SC-CO_2_. Figure 4 illustrates the juice processing.

The treatment of cane juice with direct injection of SC-CO_2_ was carried out in a 100 mL-reactor of a supercritical fluid system (Thar Technologies SFE-500, Pittsburgh/USA) available at the Laboratory of High Pressure Technology and Natural Products. The juice sample (100 mL) was transferred to the reactor and kept under the pre-set conditions (subsequently described). At the end of the treatment the sample was moved out through rapid depressurization to a sterilized glass flask.

Table 6 points out the independent variables and their actual and coded levels tested according to Rodrigues and Iemma^29^. Pressures (P) in the range of 74 to 351 bar, temperatures (T) between 33 and 67 °C, and holding times (t) varying from 20 to 70 min were tested in a central composite rotatable design. Seventeen trials were performed. This study aimed at exploring a wide range of CO_2_ pressure (above the critical one – 73.8 bar). Also the operational limits of the equipment available to conduct this study were considered in the range of the investigated parameters set. Because no study carried out with cane juice was found, no reference is herein mentioned. As for the temperature, mild values were targeted to preserve the original quality of the juice.

To get an approximate statistical inference, three trials were conducted at the central point of the experimental space; they can provide valuable information on the behavior of the responses between the levels attributed to the factors, and demonstrate the repeatability of the process (Rodrigues and Iemma)^29^.

The physicochemical, enzymic, microbiological analysis and instrumental determination of color parameters were carried out on raw and processed samples to evaluate the performance of multiple combinations of the processing’s parameters (pressure, temperature and holding time) and are as follows. All assays were performed in triplicate as described in Petrus and Simões^30^.

The physicochemical tests were performed according to the Association of Official Analytical Chemists (AOAC, 2010)^31^. An Analyzer model 300 M was used to determine the pH. The soluble solids content (expressed in °Brix) was determined in a Reichert model AR 200 portable digital refractometer.

Counts of mesophiles, molds and yeasts, lactic bacteria, and coliforms (at 45 °C) were conducted following the protocol described in the Compendium of Methods for the Microbiological Examination of Foods (Salfinger and Tortorello)^32^.

The protocols adapted from ref. ^33^ were used to determine the polyphenol oxidase (PPO) and peroxidase (POD) activities.

Five and half milliliters of 0.2 M phosphate buffer solution (pH 6.0) and 1.5 mL of 0.2 M catechol were added into a test tube and maintained at 25 °C for 10 min. Then 1.0 mL of the diluted sample in deionized water (1:10) was added. The tube was stirred for 15 s and returned to the water bath at 25 °C for 30 min. The absorbance was read in a spectrophotometer at 425 nm. The blank was prepared by diluting the sample in deionized water.

Seven milliliters of 0.2 M phosphate buffer solution (pH 5.5) and 1.0 mL of the diluted sample (juice) in deionized water (1:10) were added to a test tube and maintained in a heat bath at 35 °C for 10 min. Then 1.5 mL of 0.05% guaiacol and 0.5 mL of 0.1% hydrogen peroxide were added. The tube was magnetically stirred for 15 s and returned to the bath at 35 °C for 15 min. Finally, the absorbance was read in a spectrophotometer at 470 nm. One (1) unit of enzyme activity (U) was defined as the amount of enzymic extract capable of increasing absorbance at 425 and 470 nm for PPO and POD, respectively, at rates of 0.001 units per minute.

The color parameters (L*, a* and b*) of unprocessed and treated juice’s samples were measured in a Hunterlab Ultra-Scan colorimeter (Hunter Associates Laboratory, Model SN7877 Reston, VA/USA). The iluminant D65 and observation angle at 10° were set up. The parameters a* and b* were used to determine chroma (C) and hue angle (°hue) (Eq. 3 and 4). To compare the raw juice to the processed one, total color difference (TCD) was calculated by Eq. 5. Also L*, a* and b* were inserted in the EasyRGB to convert them into color image (EasyRGB)^34^.

3 $$C={\left({a}^{\ast 2}+{b}^{\ast 2}\right)}^{\frac{1}{2}}$$

4 $$^\circ hue=arctan\left(\frac{{b}^{\ast }}{{a}^{\ast }}\right)$$

5 $$TCD={\left(\varDelta {L}^{\ast 2}+\varDelta {a}^{\ast 2}+\varDelta {b}^{\ast 2}\right)}^{\frac{1}{2}}$$

L*: lightness (0 a 100).

a*: coordinate red (+60) / green (−60).

B: coordinate yellow (+60) / blue (−60).

ΔL*: lightness variation

Δa*: red/green variation

Δb*: yellow/blue variation

Data from the central composite rotatable design were first subjected to the analysis of effects to identify the variable(s) (P, T and t) that had significant effect on the responses (PPO, POD, coliforms, mesophiles, molds and yeasts, and lactic bacteria reduction, total color difference, pH and soluble solids variation), at 10% of significance. Due to the high variability of processes involving microorganisms and enzymes, *p*-values below 10 percent (*p* ⩽ 0.1) are considered significant parameters, as stated by Rodrigues and Iemma (2015). The analysis of regression was performed for both 1st and 2nd (responses including axial points) orders. Then the mathematical model was re-parameterized considering only the statistically significant coefficients. The analysis of variance (ANOVA) was undertaken to evaluate if the model was statistically significant. If so, the response surface was generated. Statistical tests were performed using the software Protimiza Experimental Design (http://experimental-design.protimiza.com.br).

### Reporting summary

Further information on research design is available in the Nature Research Reporting Summary linked to this article.

### Supplementary information

Reporting summary

## Data Availability

The datasets generated during and/or analyzed during the current study are available from the corresponding author upon reasonable request.
